# Application of high-frequency ultrasound in the diagnosis of gastrointestinal magnet ingestion in children

**DOI:** 10.3389/fped.2022.988596

**Published:** 2023-01-13

**Authors:** Yue Xin, Li Qun Jia, Ya Wei Dong, Yu Wang, Yan Xiu Hu, Xiao Man Wang

**Affiliations:** Department of Ultrasound, National Center for Children’s Health, Beijing Children’s Hospital, Capital Medical University, Beijing, China

**Keywords:** children, magnet, gastrointestinal, ultrasound, foreign body

## Abstract

**Background:**

The incidence of magnet ingestion by children has recently increased in China. Magnet ingestion is associated with an extremely high risk of gastrointestinal damage because loops of bowel can become trapped and squeezed between multiple magnets in different locations. However, the lack of imaging sensitivity makes clinical decision-making difficult.

**Objective:**

This study was conducted to investigate the performance of ultrasound in diagnosing gastrointestinal magnet ingestion in children.

**Methods:**

From April 2017 to February 2021, all children with a history of magnet ingestion or a diagnosis of gastrointestinal magnet as shown by x-ray or ultrasound in our hospital were included as study candidates. Patients who were lost to follow-up or had known malformations of the gastrointestinal tract were excluded. Eligible patients were those with surgical or endoscopic confirmation of gastrointestinal magnet, those who passed the magnet out of the alimentary tract without assistance, and those with confirmed absence of the magnet on abdominal x-ray examination after 1 month of conservative treatment. All eligible patients' ultrasound and x-ray examination data were evaluated. The sensitivity, specificity, and area under the curve (AUC) of ultrasound was calculated for diagnosing magnet ingestion, locating the magnet (stomach, small intestine, or colon), and confirming the phenomenon of wall entrapment.

**Results:**

Of 112 patients, 107 had a magnetic foreign body and 5 did not. Magnets were correctly detected by ultrasound in 97 patients, with an observed sensitivity of 90.65% and specificity of 100%. Satisfactory sensitivity was obtained for ultrasound localization of gastric magnets (96.30%) and small intestinal magnets (100.00%), but sensitivity for ultrasound localization of colonic magnets was relatively poor (73.33%). The discrimination of wall entrapment by ultrasound was good (AUC = 0.93), with an observed sensitivity and specificity of 92.00% and 93.62%, respectively.

**Conclusions:**

Ultrasound can be used to locate gastrointestinal magnets (in the stomach, small intestine, or colon) with good clinical efficacy in identifying wall entrapment.

## Introduction

Gastrointestinal foreign bodies are commonly seen in children, but most (80%–90%) cause no harm and pass out of the alimentary tract without complications (1–3). However, magnetic foreign bodies are special; multiple magnets can attract each other, which causes particular concerns if they are located in different gastrointestinal regions. In such cases, the magnets can trap and squeeze the gastrointestinal wall, resulting in ischemia, pressure necrosis, perforation, internal fistulas, and other serious complications (4–22). Additionally, multiple magnets do not separate (7, 23) or pass out of the alimentary tract without assistance.

Buckyballs are a new type of toy made of magnets and have recently become popular among children. However, they are small and brightly colored, resembling candy (24), and are thus easy for children to swallow. Buckyballs can also attract each other through multiple layers of the gastrointestinal wall, leading to wall injuries as described in a series of literature (2, 6, 7, 24, 25). Notably, almost all imaging examinations in these studies were x-ray examinations.

The incidence of magnet ingestion by children has recently increased in China (2). We have great experience in using high-frequency probes to explore the gastrointestinal tract of children, and we previously used such probes to evaluate colonic polyps (26) and Meckel's diverticulum (27), which are widely recognized by clinicians. In the present study, we retrospectively analyzed children with gastrointestinal magnetic foreign bodies who were diagnosed by endoscopy, surgery, or clinical follow-up in our hospital, and we discuss the value of high-frequency ultrasound in providing imaging evidence for clinical decision-making.

## Materials and methods

This retrospective study was approved by our Medical Ethics Committee (Approval No. 2021-E-185-R) and was conducted in accordance with the Declaration of Helsinki. Because all information that could identify the patients was removed, the requirement for informed patient consent for this retrospective study was waived by the Medical Ethics Committee.

### Patients

From April 2017 to February 2021, all children with either a history of magnet ingestion or relevant symptoms and confirmation of gastrointestinal magnets as shown by imaging examination (x-ray or ultrasound) in our hospital were included as study candidates. A final diagnosis was made by surgery or endoscopy. For children receiving conservative treatment, the final diagnosis was made when the magnetic foreign body passed out of the alimentary tract without assistance or was no longer visible by abdominal x-ray during the 1-month follow-up. All children underwent ultrasound examination. Patients lost to follow-up or with known malformations of the gastrointestinal tract were excluded from the study. Clinical data, x-ray findings, and ultrasound findings were reviewed.

### Ultrasound scan

Children who did not cooperate were administered 10% chloral hydrate at 0.5 ml/kg orally as a sedative and underwent an ultrasound examination when calm. There were no special fasting requirements.

A VISION Ascendus ultrasound system (Hitachi Healthcare, Tokyo, Japan) with an L52 probe (3–7 MHz) and an L74 probe (5–13 MHz) or a Philips iU22 ultrasound system (Philips Medical Systems, Bothell, WA, USA) with an L12–5 probe (5–12 MHz) and a C8–5 probe (5–8 MHz) were used for the ultrasound examinations.

**Figure 1:**
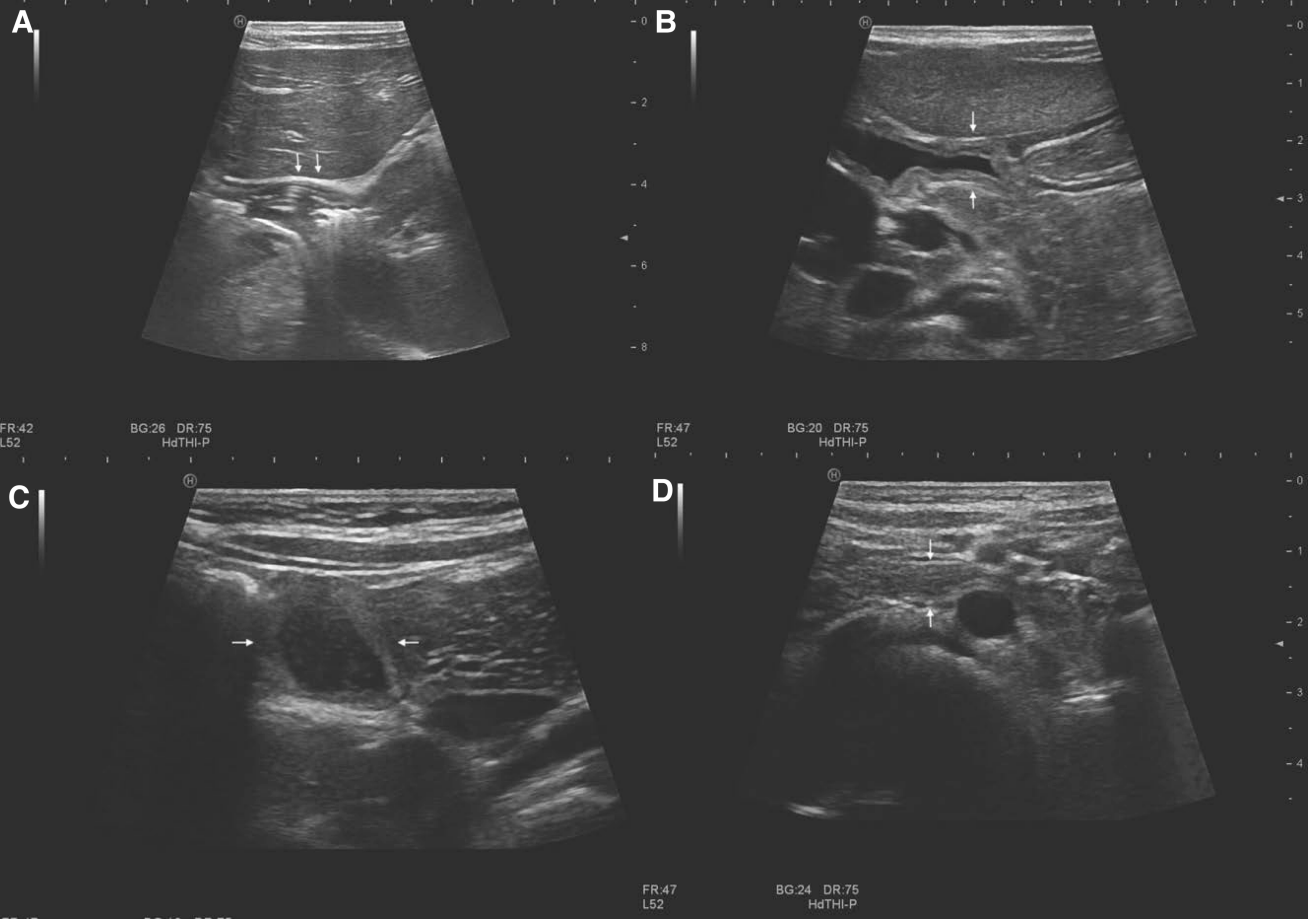
Normal gastrointestinal sonographic images obtained with high-frequency probes in a 2-year-old girl without magnets. Sonograms display the cardia (arrow in **A**), duodenal bulb (arrow in **B**), descending portion of duodenum (arrow in **C**), and horizontal portion of the duodenum (arrow in **D**).

**Figure 2:**
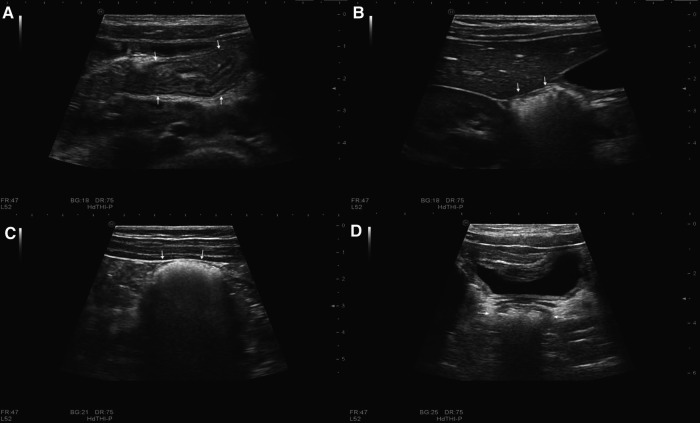
Normal gastrointestinal sonographic images obtained with high-frequency probes in a 5-year-old girl without magnets. Sonograms display the small intestine (arrow in **A**), hepatic flexure of the colon (arrow in **B**), descending colon (arrow in **C**), and rectum (arrow in **D**).

An ultrasound specialist with at least 5 years' experience in pediatric gastrointestinal sonography performed the ultrasound examinations. Gastrointestinal scanning involved placement of the probe longitudinally under the xiphoid process to identify the cardia (Figure 1A) and observe the stomach. The probe was then placed diagonally under the xiphoid process and tilted toward the right epigastric region to identify the duodenal bulb (Figure 1B) connected to the pylorus. Moving down along the duodenal bulb, the descending portion of the duodenum (Figure 1C) was lateral to the head of the pancreas and the horizontal portion (Figure 1D) were located between the superior mesenteric artery and the abdominal aorta. The remainder of the small intestine (Figure 2A) was then examined throughout the entire abdomen.

Next, the probe was placed in the right lower abdomen to locate the ileocecal section, which showed a characteristic mushroom head appearance (26), and the site of the cecum was confirmed. From here, the ascending colon was located up toward the right upper abdomen. The hepatic flexure (Figure 2B) and the splenic flexure of the colon were confirmed to be surrounded by the spleen, liver, and kidney (26), with the transverse colon between them. Moving down from the splenic flexure of the colon, the descending colon (Figure 2C) was located down toward the left lower abdomen. The rectum (Figure 2D) was located behind the bladder, and the sigmoid colon was confirmed to be located between the rectum and the descending colon.

**Figure 3:**
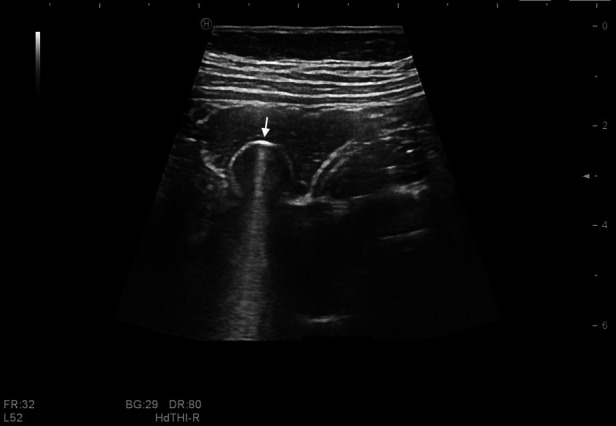
Single magnet in the gastrointestinal tract of a 2-year-old boy. A strong arc-shaped echo with a comet tail sign (white arrow) is seen in the ascending colon, with a diameter of 1.2 cm.

**Figure 4:**
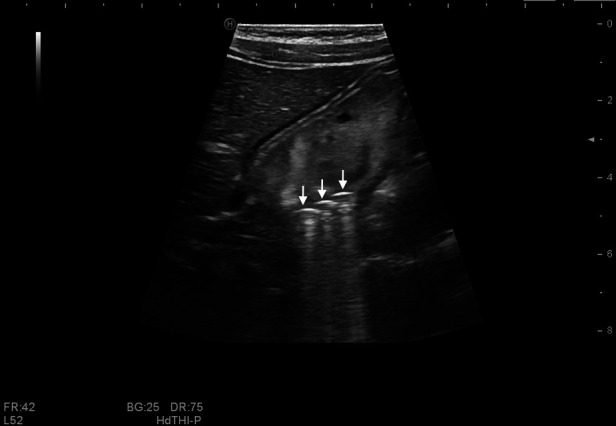
Multiple magnets in the stomach of an 11-year-old boy. Three strong, arc-shaped echoes with comet tail signs (white arrow) are seen in the stomach, with a diameter of 5 mm. No wall entrapment was found.

**Figure 5:**
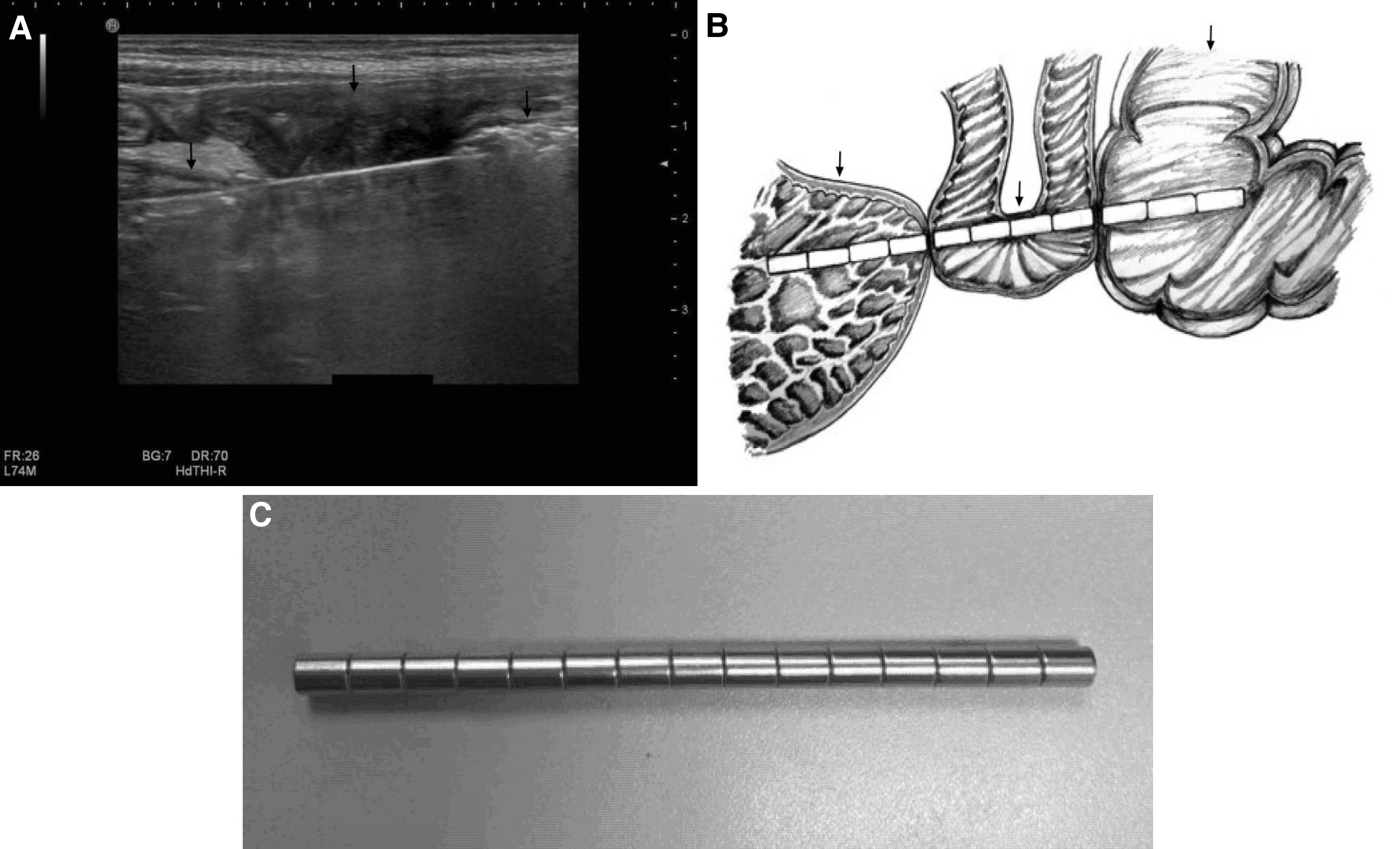
Multiple magnets in the gastrointestinal tract of a 2-year-old boy. A strong, long linear-shaped echo with a comet tail sign, representing multiple magnets attracted to one another, is seen in the stomach (left black arrow), small intestine (middle black arrow), and colon (right black arrow). (**A**) This indicates wall entrapment, which was confirmed by surgery. A further hand-drawn schema is presented to illustrate the position and surrounding conditions of the magnets. (**B**) Photograph of multiple magnets taken during surgery (**C**).

**Figure 6:**
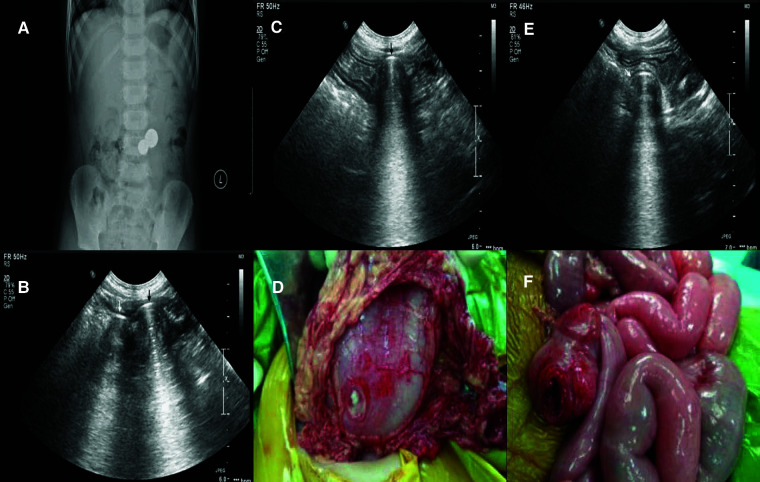
Multiple magnets in the gastrointestinal of a 6-year-old boy. The plain film indicated two magnets attracted together (**A**), as indicated by the sonogram. (**B**) The sonogram revealed one magnet (black arrow) in the stomach (**C**) and the other magnet (white arrow) in the colon. (**E**) This indicates wall entrapment, which was confirmed by surgery. Perforation of the stomach (**D**) and colon (**F**) were found during surgery.

The ingested magnets showed a sharp, strong echo accompanied by a comet tail sign (Figures 3, 4). Most appeared as arc or linear shapes depending on the form of the foreign body. The specific location of the magnets (within the stomach, small intestine, or colon) was determined. Children who had ingested multiple magnets were evaluated for the phenomenon of wall entrapment (Figures 5, 6, Supplementary Videos S1, S2), which was defined as trapping and squeezing of the gastrointestinal wall by magnets showing attraction to each other in different gastrointestinal regions.

If the first specialist failed to identify a magnet, a second ultrasound specialist with at least 10 years' experience in pediatric gastrointestinal sonography repeated the ultrasound examination. If neither specialist identified a magnet, it was considered that no magnet was present.

### Statistical analysis

SPSS software version 22.0 (IBM, Armonk, NY, USA) was used for statistical analysis. Quantitative data are expressed as median and quartiles, and qualitative data are expressed as ratio or percentage. The sensitivity, specificity, area under the curve (AUC), 95% confidence interval (CI), positive likelihood ratio, and negative likelihood ratio were used to evaluate efficacy using MedCalc Statistical Software version 19.1 (MedCalc Software Bvba, Ostend, Belgium). A *P* value of <0.05 was considered statistically significant.

## Results

### Patients

In total, 112 children were included in this study (68 boys and 44 girls) with a median age of 51.5 months (interquartile range, 29.5–79.3 months). Among them, no magnet was confirmed in 5 children, a gastrointestinal magnet was confirmed in the remaining 107 children by surgery, endoscopy, or during follow-up in our hospital. Of these 112 children, 92 (82.1%) had a history of magnet ingestion ranging from 1 h to 30 days previously, and 64 (57.1%) were asymptomatic. The clinical symptoms in the remaining 48 children included abdominal pain (*n* = 34, 50.7%), vomiting (*n* = 26, 38.8%), fever (*n* = 4, 6.0%), crying (*n* = 2, 3.0%), and abdominal distension (*n* = 1, 1.5%) (Table 1).

**Table 1:** Clinical characteristics and management of 112 patients.

Age (mo)	
Medians	51.5
Interquartile range	29.5–79.3
Sex (*n*)	
Male	68
Female	44
Clinical manifestation (*n*)	
Abdominal pain	34
Vomiting	26
Fever	4
Crying	2
Abdominal distention	1
No clinical manifestation	64
Magnet (*n*)	
Single	7
Multiple	100
None	5
x-ray results (*n*)	
Positive^a^	107
Negative	5
The results of intervention or follow-up for the patients with magnets (*n*)	
Endoscope only	14
Surgery only	45
Endoscope combined with Surgery	4
Pass out of the alimentary tract without assistance	44

^a^ The magnets were located in the umbilical region in 54 patients, hypogastric region in 23, epigastric region in 16, left hypochondriac region in 6, right iliac region in 4, right lumbar region in 2, and left hypochondriac region in 2.

A single magnet (Figure 3) was detected in 7 patients, and multiple magnets were found in 100. The magnets passed out of the alimentary tract without assistance in 44 patients. The magnets were removed by intervention in the remaining 63 patients: by endoscopy in 14, surgery in 45, and combined endoscopy and surgery in 4 (Table 1).

### Imaging evaluation

Among all 112 cases, the sensitivity of x-ray and ultrasound in diagnosing the presence of gastrointestinal magnets was 100% (95% CI, 96.61%–100.00%) and 90.65% (95% CI, 83.48%–95.43%), respectively. The specificity was 100% (95% CI, 47.82%–100.00%) for both x-ray and ultrasound. The AUC, positive likelihood ratio, and negative likelihood ratio for x-ray were 1.00 (95% CI, 0.97–1.00; *P* < 0.001), ∞, and 0.00, respectively, and those for ultrasound were 0.95 (95% CI, 0.90–0.98; *P* < 0.001), ∞, and 0.09 (95% CI, 0.05–0.17), respectively (Table 2).

**Table 2:** Diagnostic performance between different examinations of 112 patients.

	Ultrasound	x-ray
Prevalence, %	95.54 [107/112]
Diagnostic result^a^		
Magnet	97	107
No magnet	15	5
Diagnostic performance		
Area Under Curve	0.95 (0.90–0.98)	1.00 (0.97–1.00)
Sensitivity, %	90.65 (83.48–95.43) [97/107]	100 (96.61–100.00) [107/107]
Specificity, %	100 (47.82–100.00) [5/5]	100 (47.82–100.00) [5/5]
Positive Likelihood Ratios (C)	∞	∞
Negative Likelihood Ratios (C)	0.09 (0.05–0.17)	0.00

Data in parentheses: 95% confidence interval. Data in brackets: raw data.

(C), conventional.

^a^ Raw data.

Among the 107 children confirmed to have gastrointestinal magnets, x-ray indicated that 54 had magnets located in the umbilical region, 23 in the hypogastric region, 16 in the epigastric region, 6 in the left hypochondriac region, 4 in the right iliac region, 2 in the right lumbar region, and 2 in the left hypochondriac region (Table 1).

Among the 97 children with magnets identified by ultrasound, the AUC of wall entrapment was 0.93 (95% CI, 0.86–0.97; *P* < 0.001), with an observed sensitivity of 92.00% (95% CI, 80.77%–97.78%) and specificity of 93.62% (95% CI, 82.46%–98.66%). The positive and negative likelihood ratios were 14.41 (95% CI, 4.81–43.21) and 0.09 (95% CI, 0.03–0.22), respectively.

Among the 63 children diagnosed by endoscopy or surgery, the ultrasound findings of the specific locations of the magnets are shown in Table 3. The sensitivity of ultrasound in localizing gastric and small intestinal magnets was 96.30% (95% CI, 81.03%–99.91%) and 100.00% (95% CI, 92.13%–100.00%), respectively. However, the sensitivity of detecting colonic magnets was relatively poor at 73.33% (95% CI, 44.90%–92.21%). The specificity of ultrasound in localizing gastric and colonic magnets was 100% (95% CI, 92.60%–100.00%) for both, which was higher than that for detecting small intestinal magnets, at 88.89% (95% CI, 65.29%–98.62%).

**Table 3:** Diagnostic performance of location indicated by ultrasound of 63 patients.

Location	Final diagnosis (*n*)	Diagnostic performance of ultrasound	
		Sensitivity, %	Specificity, %
Stomach	27	96.30 (81.03–99.91) [26/27]	100 (90.26–100.00) [36/36]
Small intestine	45	100.00 (92.13–100.00) [45/45]	88.89 (65.29–98.62) [16/18]
Colon	15	73.33 (44.90–92.21) [11/15]	100 (92.60–100.00) [48/48]

Data in parentheses: 95% confidence interval. Data in brackets: raw data.

## Discussion

The present study showed that the discrimination of high-frequency ultrasound in indicating the presence of gastrointestinal magnetic foreign bodies was good (AUC = 0.95), with an observed sensitivity and specificity of 90.65% and 100%, respectively. Satisfactory sensitivity was obtained for detection of gastric and small intestinal magnets by ultrasound (96.30% and 100.00%, respectively), although the sensitivity of detecting colonic foreign bodies was relatively poor at 73.33%; this may reflect interference by feces and gas in the colon. The specificity of ultrasound in detecting gastric and colonic magnets was 100% in both cases, which is higher than the 88.89% seen for small intestinal magnets.

We believe that sensitivity is more important than specificity for the detection of magnetic foreign bodies in the gastrointestinal tract. Once multiple magnets have been detected in the gastrointestinal tract, the complication of wall entrapment can be evaluated to provide imaging evidence for further clinical diagnosis and treatment. We observed good discrimination of wall entrapment by ultrasound (AUC = 0.93), with a sensitivity and specificity of 92.00% and 93.62%, respectively. Thus, ultrasound was a useful supplement to x-ray in indicating wall entrapment.

Although a single small magnet does not cause damage to the gastrointestinal tract (4, 28), the best method of managing ingestion of multiple magnets remains under discussion. It is recommended that such children are evaluated by a clinician, but the management principle will depend on the location of the magnets and the presence of clinical symptoms. Vigilance is needed whether plain abdominal films show changes in the magnet position, free gas beneath the diaphragm, or intestinal obstruction (7). Among the 97 children in whom gastrointestinal magnets were indicated by ultrasound in the present study, only 3 had ingested a single magnet; the remaining 94 had ingested multiple magnets. Multiple magnets can attract each other, which is particularly dangerous if the magnets are located in different gastrointestinal regions. In a previously reported case of multiple magnet ingestion, ulceration and indentation of the mucosa occurred within 8 h (7). Gastrointestinal perforation caused by magnets usually has no typical clinical or radiographic manifestations (29); thus, the onset of symptoms in children can be late, or the children can even be asymptomatic. In the present study, five children were found to have gastrointestinal perforation during surgery, but all were asymptomatic. By the time a child shows typical symptoms and x-ray examination reveals gastrointestinal perforation, the intestinal wall may already be seriously damaged. Of the 49 children who underwent surgical treatment in our study, gastrointestinal injury was found in 46 (93.9%). Among the 46 children, ischemia and necrosis of the gastrointestinal wall was found in 4, gastrointestinal mucosal erosion or ulceration in 4, and gastrointestinal perforation in 38. Among the 38 children with gastrointestinal perforation, a concurrent fistula was present in 13 and concurrent intestinal obstruction was present in 12. However, it is not feasible to blindly intervene or to expose children to unnecessary costly treatments. Among the 100 patients with confirmed multiple magnets in our study, 32 children were found to have no wall entrapment by ultrasound, and the magnets in these 32 children passed out of the gastrointestinal tract without assistance. These findings suggest the importance of determining whether the magnets have attracted each other and caused wall entrapment as soon as possible after ingestion. Such confirmation by imaging can assist in timely and accurate clinical decision-making.

Plain films of the abdomen are the first-choice or even only imaging examination used to judge the presence, quantity, and approximate location of magnetic foreign bodies that have been ingested (4, 7, 30). Although the sensitivity of high-frequency ultrasound in detecting ingested magnets was lower than that of x-ray in this study, ultrasound was nevertheless able to determine the magnet location in the stomach, small intestine, or colon, and its sensitivity for localizing gastric and small intestinal magnets was good. Its sensitivity was poorer in detecting colonic magnets, but these readily passed out of the gastrointestinal tract. Of particular note, three children in the present study had gastrointestinal magnets detected by ultrasound as their first examination. Together, these findings suggest that ultrasound can be used as a complement to x-ray examination to optimize the inspection process, reduce radiation exposure, and provide new evidence to support clinical decision-making.

However, ultrasound examination also has some limitations. It is less sensitive than x-ray in identifying gastrointestinal magnets. Although high-frequency ultrasound has greatly improved imaging clarity, interference by colonic gas and feces can still lower the sensitivity of detecting colonic magnets. This may lead to misdiagnosis and missed diagnosis of wall entrapment. Therefore, we suggest that x-ray be used as the first method of examination following magnet ingestion to determine the number and approximate locations of the magnets. Ultrasound can then be used as a supplemental form of imaging to evaluate the potential risk of complications.

Our study provides a reliable imaging basis for the diagnosis and treatment of gastrointestinal magnets in children, but it still has limitations. First, this was a single-center retrospective study, and case selection bias was inevitable. Therefore, the results should be verified by prospective diagnostic tests. Second, identifying the phenomenon of wall entrapment proposed in this study depends on the objective experience of the operator, and identification of this phenomenon might benefit from the development of a scientific training program. Third, because of the small number of patients, we could not establish a complete clinical model to predict the exact probability of gastrointestinal injury based on a stratification analysis. Thus, a further study focusing on this topic is of utmost importance.

In conclusion, high-frequency ultrasound can reveal the specific location (stomach, small intestine, or colon) of gastrointestinal magnets following their ingestion by children and has good clinical efficacy in indicating the phenomenon of wall entrapment. As a complement to x-ray analysis, high-frequency ultrasound provides an important imaging basis for clinical decision-making to reduce or mitigate the potential damage caused by gastrointestinal magnets.

## Data Availability

The datasets presented in this article are not readily available because. The datasets aren't available unless the permission of corresponding author. Requests to access the datasets should be directed to Xiao Man Wang, pcchty@yeah.net.
